# Clinical and Demographic Features of Basal Cell Carcinoma in North Jordan

**DOI:** 10.1155/2018/2624054

**Published:** 2018-10-04

**Authors:** Firas Al-Qarqaz, Maha Marji, Khaldon Bodoor, Rowida Almomani, Wisam Al Gargaz, Diala Alshiyab, Jihan Muhaidat, Mohammad Alqudah

**Affiliations:** ^1^Department of Dermatology, Jordan University of Science and Technology, P.O. Box 3030, Irbid, Jordan; ^2^Department of Applied Biology, Jordan University of Science and Technology, P.O. Box 3030, Irbid, Jordan; ^3^Department of Medical Laboratory Sciences, Jordan University of Science and Technology, P.O. Box 3030, Irbid, Jordan; ^4^Department of Special Surgery, Jordan University of Science and Technology, P.O. Box 3030, Irbid, Jordan; ^5^Department of Pathology, Jordan University of Science and Technology, P.O. Box 3030, Irbid, Jordan

## Abstract

Basal cell carcinoma (BCC) is the most common cancer affecting humans. It almost has no tendency for metastasis; however it can be destructive to surrounding tissue. Patients with darker skin colors have lower risk of developing skin cancers and the clinical characteristics may differ from populations with lighter skin colors.* Methods*. This is a retrospective clinical study (2003–2017). Data on age, gender, and location of tumor were collected and analyzed.* Results*. 335 cases were identified. Males tend to get BCC at a younger age than females. Face was the most common site in both males and females. Cheeks and nose were the most likely areas of the face to be involved. Scalp was the most common extrafacial site to be involved in males; however in females scalp was much less likely to be involved.* Conclusion*. BCC is less common in populations with darker skin. Males were more affected and at an earlier age compared to females. Facial skin followed by scalp was the most common site affected. Skin phototype, cultural and religious dress type, and different sun exposure behavior may explain many of the clinical and demographic findings related to BCC in patients with darker skin tones.

## 1. Introduction and Background

Basal cell carcinoma (BCC) is the most common skin cancer especially in populations with fair skin color. The pathogenesis of BCC is linked to excessive sunlight exposure [1]. This explains the predominance of facial involvement by this cancer. Additional factors that increase the risk of BCC development include, among others, genetic alterations HPV infection and immune suppression [2, 3].

Despite being the most common skin cancer, BCC has almost no potential for metastasis and hence the mortality from this tumor is negligible. However, the morbidity from this slowly growing tumor can be significant due to local tissue damage [4, 5].

The incidence of BCC is on the rise worldwide; for example, in Netherlands the incidence increased by 3-fold between 1973 and 2008 from 40 to 148 per 100,000 in males and from 34 to 141 in females [6]. The true incidence is however even higher as these tumors are generally underreported.

The diagnosis of BCC is initially suspected based on the appearance of a slowly growing firm translucent nodule with surface telangiectasia and rolled margins; however there is some variation according to the type of BCC. Dermoscopic examination can provide additional important clues for diagnosis of BCC; however confirmation of diagnosis requires histopathologic examination. Comprehensive pathologic assessments, including histologic subtype, invasion behavior, among other features, are required as prognostic indicators to help predict recurrence and potential for invasion by the tumor cells [7, 8].

Several key genes have been implicated in BCC pathogenesis, especially protooncogenes and tumor suppressor genes. These include, among others, PTCH1 and SMO genes which are key components of the Hedgehog (Hh) pathway, TP53 gene, and other RAS protooncogene family members [9, 10].

There is wealth of data on BCC in Caucasian skin with lighter skin colors but much less information exist on populations with dark skin [11–14]. It is clear that skin cancer is much less common of a problem in these populations which may explain why there are less interest and data populations with darker skins.

**Figure 1:**
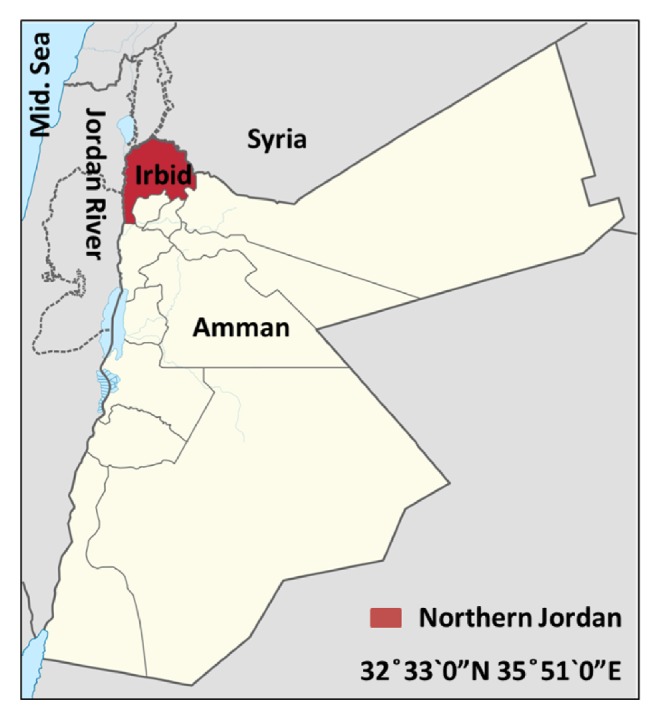
Map of Jordan showing the geography of the study area.

Northern Jordan is part of the Hawran plateau which covers northern Jordan and southwest Syria. The governorate is bordered by Syria (the Golan Heights) from the north, the Jordan River from the west, Mafraq Governorate from the east, and Jerash, Ajloun, and Balqa Governorates from the south (Figure 1). The geographic coordinates for Irbid are 32°33′0′′N 35°51′0′′E with altitude of around 620 meters. Northern Jordan has a hot-summer, Mediterranean climate with at least 4 months averaging above 30°C (June-September).

In this study we highlight the main demographic and clinical features of BCC in North Jordan population which predominantly has darker skin tones, mainly types 3 and 4.

## 2. Materials and Methods

This is a retrospective descriptive study. BCC cases were retrieved from the electronic records from the Department of Pathology at King Abdulah University Hospital in Irbid, north of Jordan, for the period 2004-2018. For the year 2003 BCC, cases were included for the calculation of the overall number but were not included in the age-adjusted incidence analysis because population data were missing from the national department of national statistics. The confirmed diagnosis of BCC at the final conclusion of pathology reports was used for the inclusion of these cases. The Pathology Department affiliated to Faculty of Medicine, Jordan University of Science and Technology, is the only pathology department for north of Jordan area with catchment area population of around 1,2 million. Clinicopathological and demographic parameters were statistically analyzed to identify significant correlations.* t*-test was used for calculation of p values using SPSS software package 17. P value was considered significant if it was less than or equal to 0.05. The study was approved by the Research Committee of the Faculty of Medicine and the IRB of the university.

## 3. Results

**Figure 2:**
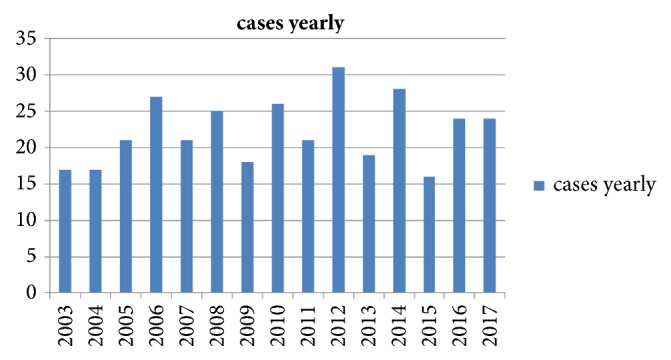
Number of BCC new cases over 15 years: 2003-2018.

A total of 335 confirmed BCC cases were identified and included in the study. The cohort included 214 males and 121 female cases with male: female ratio of 1.76: 1. The mean age for females was 65.4 years (35-91 years), while the mean age for males was 61.9 years (27-96 years). The difference between means of age for males and females was statistically significant (P = 0.018). The average number of new cases of BCC is around 22.3 (16-31) cases per year (Figure 2). There was no significant variation in number of BCC cases over the 15 years (P value 0.31).

The incidence rates of BCC in northern Jordan ranged from 1.5 to 3 per 100,000 population for the years 2004 to 2017. However because BCC is usually increased with age, we also calculated the age-adjusted incidence rates for the study population. Based on the data from the Jordanian Department of Statistics [15], the age-adjusted incidence rates for BCC were calculated for the study population based on age categories: 0-19 years, 20-39 years, 40-64 years, and 65+ years. The age-adjusted incidence rates per 100,000 population results are shown in Table 1. For the age groups 0-39 years, the incidence rates were negligible (0-0.65 per 100,000 population). For the age group 40-65 years, the rates were between 2.4 and 7.7 per 100,000 population. However for the age group above 65 years the incidence rates were higher (9-31 per 100,000 population) as shown in Table 1.

**Figure 3:**
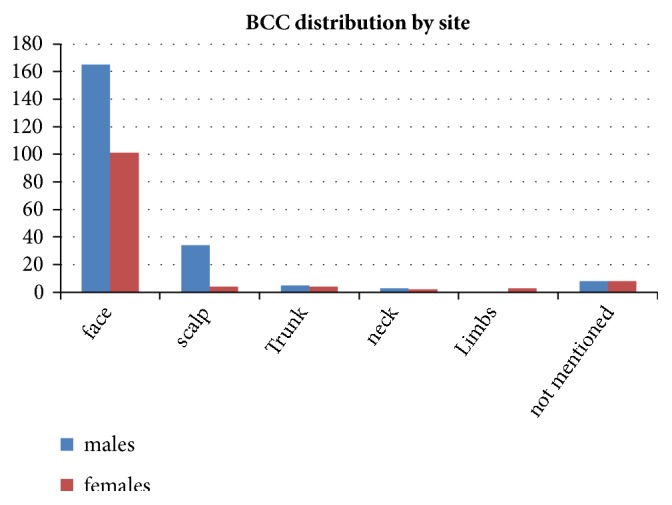
BCC distribution according to site.

In terms of tumor location, our data clearly shows that face was the most affected site in both males and females, accounting for 77% and 83% of all BCC cases, respectively (Figure 3). The most common nonfacial BCC was scalp in males accounting for 16% of cases, whereas, in females, nonfacial BCC did not show any strong predilection for specific sites with tumors scattered across various body sites (Figure 3).

Facial BCC mainly affected the centrofacial sites, namely, nose, cheeks, and eyelids skin (Figure 3). This distribution does however vary between males and females and this variation is statistically significant (p =0.01). The most important differences in distribution between males and females were related to forehead, ears, and temples being more affected in males but much less in females (18% versus 8% for these sites combined).

Of the 335 cases in this study, 12 cases (8 males and 4 females) had multiple BCCs while the remainder had only one BCC.

## 4. Discussion

The link between skin cancer and sunlight exposure is well established with increasing incidence of skin cancers in populations living near equator where there is stronger exposure to sunlight and this is especially manifested in populations with fair skin colors. However, in areas where the dominant skin color is dark, the risk for skin cancer is still generally low [16]. In a study from one center in Jordan a predominantly colored-skin population (types 3 and 4 mainly) there were 67 cases over 5 years (around 13 cases per year) [14].

In the current study, the cohort consisted mainly of skin phototypes 3 and 4 making around 82% of all cases. The annual number of new cases of BCC ranged from 16 to 31 with an average of around 22 cases. The difference over the years did not change significantly while in most countries with more fair skin color the incidence of BCC is increasing steadily [16–18].

In this population from north Jordan the overall incidence rate for BCC ranged from 1.5 to 3 per 100,000. In Saudi Arabia, a recent study has indicated incidence rates between 3.4 and 4.4 per 100,000 population [19]. Another study from Iran showed incidence rates for BCC between 10.05 and 15.57 per 100,000 population [20]. Additional studies from Jordan and neighboring countries with similar dark skin color are also in accordance with low incidence rates for BCC reported in this study [21–24]. It is worth noting that, in Caucasian skin populations, the incidence rates for BCC are much higher. For example, in Australia the incidence rate exceeds 1,000 per 100,000 population whereas in the United States the incidence rate is around 450 per 100.000 and in Europe the rate is around 100 per 100,000 [16].

The low incidence rates for BCC over the years in this population could be explained by the darker skin type, chronic rather than intermittent pattern of sun exposure, and generally clothes type covering most body parts except face and hands. Darker skin is more protected against damaging effects of ultraviolet radiation from solar light mostly due to protective effect by melanin [25, 26]. In this population, the general dress behavior pattern is more towards less exposed skin, i.e., long sleeves and long trousers, especially in the case of women who tend to cover almost all body and use head cover whenever they go out of their houses due to religious and social reasons. Additionally, the intermittent sun exposure “recreational” is not commonly seen on the contrary, and most people are chronically exposed to sunlight which implies more basal melanin production rates and hence more skin protection. Taken together, the aforementioned factors could be responsible for the low incidence of BCC in this population despite northern Jordan having sunny bright days during most of the year (average 310 sunny days/year).

In the current study, the average age at time of diagnosis was 61.9 years for males and 65.4 years for females. Males were more affected in this group than females; this is actually similar to what is reported from most countries [11, 27–29].

In terms of tumor location, face was the most common affected site in both males and females. This reflects again the importance of solar radiation as the main causative factor in the pathogenesis of this condition. Other sun-covered skin areas were much less commonly involved. As for extrafacial involvement, scalp was the next common site affected in males but not in females. Hair is an important photoprotector for the scalp and also many females in this population use head cover (scarf) whenever they are outdoors which could also explain the relative rarity of scalp involvement in women from this group.

Within the facial skin, the more sun-exposed regions were more likely to be affected with some variation between males and females. Nose, cheeks, and eyelids were the most sites affected in both sexes. In females, BCC in forehead, ears, and temples were less common than males (p= 0.01). This variation could also be explained by the head cover worn by many females in this population. Other studies have also demonstrated similar distribution of facial BCC with nose and cheeks being the more commonly involved sites [11, 30].

In this cohort, only 12 (3.6%) cases had multiple BCCs which is generally much less than reported by other studies [31, 32]. Multiple BCC is possibly linked to a number of risk factors including age, skin phototype (1 & 2), intermittent sun exposure and sunburns, history of radiotherapy, larger tumor size, truncal tumor, genetic factors, and geography of area especially high latitude [32, 33]. In this cohort, darker skin phototypes (3 & 4) are the most common, truncal BCCs were only seen in 9 patients (2.7%), the pattern of sunlight exposure is nonburning, non of these patients had prior radiotherapy treatment, and the geography of north Jordan is towards low altitude (620 meters). These factors could explain the low number of patients having multiple BCCs.

## 5. Conclusions

BCC in this population with darker skin color is much less common than in populations with fair skin color. The number of new cases did not change significantly over the past 15 years. Males are more affected and tend to be younger than females at time of diagnosis. Face is the most common site involved especially nose, cheeks, and eyelids. The darker skin color, type of clothing, and pattern of sunlight exposure are likely to explain the lower number of BCC cases and location distribution of BCC in this population.

## Figures and Tables

**Table 1:** Age adjusted incidence rates for BCC per 100,000 population (2004-2017)^*∗*^.

**Age categories**				
**Year**	0-19 (Y)	20-39 (Y)	40-64 (Y)	65+ (Y)
2004	0	0.00	4.60	20.86
2005	0	0.28	5.96	20.28
2006	0	0.27	7.71	24.62
2007	0	0.27	3.28	31.07
2008	0	0.26	5.00	30.15
2009	0	0.00	4.40	17.99
2010	0	0.24	5.55	26.15
2011	0	0.23	2.45	29.22
2012	0	0.66	6.93	19.65
2013	0	0.00	2.47	21.59
2014	0	0.00	5.52	18.24
2015	0	0.17	2.69	9.16
2016	0	0.50	3.50	13.41
2017	0	0.00	3.41	17.42

^*∗*^Population Data for the year 2003 was absent from department of statistics and hence was not represented here.

## Data Availability

The data used to support the findings of this study are available from the corresponding author upon request.
